# The relationship between pelvic floor distress and sexual function in women with urinary incontinence

**DOI:** 10.1590/1806-9282.20241846

**Published:** 2025-06-02

**Authors:** Seyhan Çankaya, Dilek Nur Uzun

**Affiliations:** 1 Selçuk University, Faculty of Health Sciences, Department of Midwifery – Konya, Turkey.

**Keywords:** Urinary urge incontinence, Urinary stress incontinence, Pelvic organ prolapse, Sexual dysfunction, Sexual activity

## Abstract

**OBJECTIVE:**

This study aimed to determine the relationship between pelvic organ prolapse distress, colorectal-anal distress, and urinary distress and sexual function in women with urinary incontinence.

**METHODS:**

This is a descriptive correlational study. The study was conducted in the urogynecology outpatient clinic of a training and research hospital in a province located in the central Anatolia region of Turkey. The study included 328 women with urinary incontinence.

**RESULTS:**

The women who participated in the study had urinary incontinence for 6.2 (SD 4.6) years. The mean total sexual function score of the women was 18.7 (SD 3.8), and they had poor sexual function. Pelvic organ prolapse distress, colorectal-anal distress, and urinary distress were significant associated factors negatively affecting the sexual function of the women (p<0.001). In addition, colorectal-anal distress was an important risk factor negatively affecting women's sexual function (p<0.001).

**CONCLUSION:**

Pelvic organ prolapse distress, colorectal-anal distress, and urinary distress negatively affect the sexual function of women with urinary incontinence.

## INTRODUCTION

Urinary incontinence (UI) is defined by the International Continence Society (ICS) as "involuntary urinary incontinence" and is a common public health problem affecting the lives of adult women worldwide^1,2^. The prevalence of UI globally varies between 13.1 and 70.9%^3^.

Women with UI may experience pelvic floor distress (urinary distress, pelvic organ prolapse distress, and colorectal-anal distress), and their sexual function may be negatively affected^4^. In addition to pelvic floor distress, women with UI face many physical, social, and psychological problems^5,6^. They may not seek solutions to their problems for many reasons such as embarrassment, perceiving it as a normal condition due to aging, thinking that there are no treatment options, and lack of knowledge^4,7^.

Sexual function is defined as the result of individual or collective behaviors and individual-specific satisfaction frequency in the stages of sexual desire, arousal, and orgasm and is considered an important parameter in the evaluation of quality of life^8^. Studies have reported that women with UI have lower sexual function scores than women without UI and have significantly decreased frequency of sexual activity and arousal abilities in the previous year^9,10^. Studies describing the relationship between pelvic floor distress (urinary distress, pelvic organ prolapse distress, and colorectal-anal distress) and sexual function in women with UI are limited in the literature. Therefore, this study aimed to determine the relationship between pelvic organ prolapse distress, colorectal-anal distress, and urinary distress and sexual function in women with UI.

## METHODS

### Study design

The study was designed as a descriptive and correlational study.

### Study setting

The study was conducted in the urogynecology outpatient clinic of a training and research hospital of a province located in the Central Anatolia Region of Turkey.

### Participants

Women presented to the hospital's urogynecology outpatient clinic, diagnosed with UI, and volunteered to participate in the study were included in the study. The inclusion criteria were being diagnosed with UI, being aged between 18 and 50 years, being married or having a partner, being heterosexual, having an active sexual life, being able to communicate in Turkish, and agreeing to participate in the study. Exclusion criteria were being pregnant, being in the first 6 months postpartum, and having a serious systemic disease (cardiac, respiratory, gastrointestinal, neurological, endocrine, etc.) or a psychiatric disease such as psychotic disorder and mental retardation that may prevent clinical interview.

### Population of the study

The population of the study consisted of women who presented to the hospital's urogynecology outpatient clinic between January 2023 and June 2023 and were diagnosed with UI.

### Sample and methodology of the research

The sample size of the study was calculated based on the mean score of the pelvic organ prolapse/urinary incontinence sexual function questionnaire in the study conducted by Berrada et al.^11^ (Mean: 28.72; SD: 6.9) using the G. Power-3.1.9.4 (Ver. 3.1.9.2, Franz Faul, Universität Kiel, Germany) program with an effect size of 0.2 within 1-point deviation, 5% margin of error, and 95% power, and it was determined that 328 women should be included in the sample^12^. Data were collected by means of a convenience sampling method.

### Procedure

After a detailed urogynecological examination, all women diagnosed with UI were interviewed in a private room in the urogynecology outpatient clinic and the data were collected by the researchers through face-to-face interviews. In the first interview with the women, the purpose of the study was explained, and written consent forms were obtained. They were informed that participation in the study was voluntary. No incentive was paid to the participants.

### Measures

Data were collected using a personal information form, the Pelvic Floor Distress Inventory-20 (PFDI-20), and the Pelvic Organ Prolapse/Urinary Incontinence Sexual Function Questionnaire (PISQ-12).

#### Personal information form

The form developed by the researcher in line with the literature review consists of 25 items^13^.

#### Pelvic Floor Distress Inventory-20

PFDI-20 was developed for the evaluation of pelvic floor dysfunction. The Turkish validity and reliability study of the scale was performed^14^. The scale consists of 20 items and 3 subscales: (1) Pelvic Organ Prolapse Distress Inventory 6 (POPDI-6), (2) Colorectal-Rectal Anal Distress Inventory 8 (CRAD-8), and (3) Urinary Distress Inventory 6 (UDI-6). The internal consistency reliability coefficient of the scale is 0.80, 0.73, and 0.66 for the three subscales POPDI-6, CRAD-8, and UDI-6, respectively, and 0.79 for the whole scale^14^. Individuals are asked to answer the questions as "no" or "yes" according to the presence of the complaint, and those whose answer is "yes" rate the severance of the complaint as 1–4. Each sub-factor score ranges between 0 and 100, and the total score of the scale ranges between 0 and 300. There are no reverse-scored items in the scale. The total score for pelvic floor distress is obtained by summing the 20 items in the scale. In addition, UDI-6, CRAD-8, and POPDI-6 scales can be scored separately. As the score of the questionnaire increases, the degree of complaint of pelvic floor dysfunction increases^14^. In this study, Cronbach's alpha internal consistency reliability coefficient was 0.73, 0.79, and 0.71 for POPDI-6, CRAD-8, and UDI-6 subscales, respectively, and 0.87 for the whole scale.

#### Pelvic Organ Prolapse/Urinary Incontinence Sexual Questionnaire

The PISQ-12 aims to assess sexual function specific to urogynecology and consists of 12 items in total^12^. The PISQ-12 evaluates sexual functioning in the last 6 months^15^. The PISQ-12 has three subdimensions: behavioral–emotional, physical, and partner-related^15^. The Turkish validity and reliability study of the scale was conducted, and the Cronbach alpha internal consistency reliability coefficient was calculated as 0.92 for the behavioral/emotional state subdimension, 0.89 for the physical state subdimension, 0.67 for the partner-related state subdimension, and 0.89 for the whole scale. Questions 0–4 in the form are scored. A minimum score of 0 and a maximum score of 48 can be obtained from the questionnaire. A high PISQ-12 scale score means a high level of well-being in sexual functions^15^. In this study, Cronbach's alpha internal consistency reliability coefficient of the scale was 0.76 for the behavioral/emotional state subdimension, 0.72 for the physical state subdimension, 0.70 for the partner-related state subdimension, and 0.81 for the whole scale.

### Ethical considerations

We obtained research ethics committee approval from both the local ethical board prior to the study (Ethics permit no: removed for blind review) and an institutional permit from the relevant hospital (Institution approval number: removed for blind review). All procedures were carried out in accordance with the principles of the Helsinki Declaration.

### Data analysis

The NCSS (Number Cruncher Statistical System) 2020 Statistical Software (NCSS LLC, Kaysville, Utah, USA) was used for statistical analyses while evaluating the findings obtained in the study. While evaluating the study data, quantitative variables are presented as mean, standard deviation, median, min, and max values, and qualitative variables are presented as descriptive statistical methods such as frequency and percentage. The Shapiro-Wilks test and box plot graphs were used to evaluate the suitability of the data for normal distribution. Pearson correlation analysis was used to evaluate the relationships between variables. As a multivariate analysis, the effects of the POPDI-6 subscale score, the CRAD-8 subscale score, and the UDI-6 subscale scores on Pelvic Organ Prolapse/Urinary Incontinence Sexual Function Scale (PISQ-12) score were evaluated by linear regression analysis with enter method. The confidence interval was set at 95% and significance was set at p<0.05^16^.

## RESULTS

The personal characteristics of the women are given in Table 1. The women's age ranged between 20 and 49 years, with an average age of 36.5 years (SD 7.4). All participants were married and had an average of 19.8 (SD 8) years of marriage. The mean total score of POPDI-6 was 20.3 (SD 19.3), CRAD-8 30 (SD 21.2), UDI-6 41.7 (SD 21.3), and PFDI-20 91.4 (SD 53). The mean total score of the PISQ-12 was 18.7 (SD 3.8).

**Table 1:** Personal characteristics of women (n=328).

Characteristics		Mean (SD)	Median (min–max)
BMI		30.1 (5)	29.8 (19.2–52.7)
Number of births		2.72 (1.38)	3 (0–8)
Weekly number of sexual intercourses		2.2 (1.1)	2 (1–7)
Duration of UI complaint		6.2 (4.6)	5 (1–22)
Last mode of delivery		**n**	**%**
	Vaginal delivery	102	31.1
	Caesarean section	116	35.4
	Operative vaginal delivery	110	33.5
Pelvic floor exercise status			
	Yes	67	20.4
	No	261	79.6
Frequent urinary tract infections			
	Yes	138	42.1
	No	190	57.9
Legislation of frequent vaginal infections			
	Yes	151	46
	No	177	54
Urinary and genital system operations			
	Yes	48	14.6
	No	280	85.4
Visiting a health institution for UI			
	Yes	89	27.1
	No	239	72.9
	Not knowing where to go	26	10.9
	Thinking that it is normal after vaginal birth (normalization)	133	55.6

SD: standard deviation; BMI: body mass index; UI: urinary incontinence.

Table 2 presents the correlation coefficients of PFDI-20 total and subscale scores and PISQ-12 and its subscales. A weak and moderate positive correlation was found between women's PFDI-20 total score, POPDI-6, CRAD-8, UDI-6, and PISQ-12 total score and physical status and partner-related status subscales (p<0.001, Table 2). In addition, a moderate, negative correlation was found between the PISQ-12 subdimension of behavioral/emotional state and POPDI-6, CRAD-8, UDI-6, and PFDI-20 total score (p<0.001, Table 2).

**Table 2:** Relationship between Pelvic Floor Distress Inventory-20 total and subscale scores and Pelvic Organ Prolapse/Urinary Incontinence Sexual Questionnaire and subscale scores.

Scores		Total PISQ-12	Behavioral/emotional state*	Physical condition*	Partner-related status*
Total PFDI-20	r	0.300	-0.509	0.594	0.297
	p	**<0.001**	**<0.001**	**<0.001**	**<0.001**
POPDI-6	r	0.204	-0.393	0.447	0.230
	p	**<0.001**	**<0.001**	**<0.001**	**<0.001**
CRAD-8	r	0.316	-0.452	0.595	0.268
	p	**<0.001**	**<0.001**	**<0.001**	**<0.001**
UDI-6	r	0.238	-0.448	0.517	0.258
	p	**<0.001**	**<0.001**	**<0.001**	**<0.001**

r: Pearson Correlation Test, n=328. PFDI-20: Pelvic Floor Distress Inventory-20; POPDI-6: Pelvic Organ Prolapse Distress Inventory 6; CRAD-8: Colorectal-Anal Distress Inventory 8; UDI-6: Urinary Distress Inventory 6; PISQ-12: The Pelvic Organ Prolapse/Urinary Incontinence Sexual Questionnaire.

* Subdimensions of PISQ-12. Bold value: statistically significant values (p<0.001).

The linear regression analysis according to the factors that may affect the sexual function status of women with UI is shown in Table 3. Enter linear regression analysis was performed to evaluate the effect of the three independent variables determined to be related in the correlation analysis on the sexual function of women. The regression model for risk factors that may affect women's sexual function was significant (F=12.464, p<0.001) and explained 9.5% of the variance (Table 3). In light of the regression analysis findings, colorectal-anal distress (CRAD-8) was a significant risk factor that negatively affected women's sexual function (p<0.05, Table 3).

**Table 3:** Linear regression analysis according to factors affecting sexual function status of women with urinary incontinence.

Variables	PISQ-12						
	B	SE	β	T	p	95.0%CI for B	
						Low value	High value
POPDI-6	0.003	0.014	0.017	0.246	0.806	-0.024	0.030
CRAD-8	0.047	0.012	0.264	3.865	**<0.001**	0.023	0.070
UDI-6	0.013	0.013	0.071	0.986	0.325	-0.013	0.038

POPDI-6, Pelvic Organ Prolapse Distress Inventory 6; CRAD-8, Colorectal-Anal Distress Inventory 8; UDI-6, Urinary Distress Inventory 6; PISQ-12, The Pelvic Organ Prolapse/Urinary İncontinence Sexual Questionnaire; CI: confidence interval; SE: standard error. n=328; r=0.322, r^2^=0.104, Adjusted r^2^=0.095, F=12.464, p<0.001. Bold value: statistically significant values (p<0.05).

## DISCUSSION

This study aimed to determine the relationship between pelvic organ prolapse distress, colorectal-anal distress, and urinary distress and sexual function in women with UI. To the best of our knowledge, there is limited research evaluating the relationship between pelvic floor distress and sexual function in women with UI. We think that this study makes an important contribution to the sexual health literature.

In this study, pelvic organ prolapse distress negatively affected the sexual function of women with UI. Research shows that pelvic floor disorders have a negative impact on sexual function, but it is often unclear whether pelvic organ prolapse or UI causes the most damage. Some authors report that pelvic organ prolapse is more likely to cause sexual problems than UI, while others report that decreased libido is more pronounced in women with UI than in women with pelvic organ prolapse^17^.

In this study, colorectal-anal distress was an important associated factor negatively affecting the sexual function of women with UI and was also an associated risk factor in regression analysis. Regarding bowel function, previous studies have similarly shown that constipation, difficulty in defecation, and fecal incontinence commonly occur in women with lower urinary tract symptoms^18,19^.

Another finding in this study was that women with UI or experiencing UI distress had poor sexual function. In a Swedish cross-sectional study of 147 sexually active women with UI or overactive bladder, women with UI were dissatisfied with their sex lives, with a negative impact on sexual desire and/or sexual satisfaction related to inadequate vaginal lubrication, unsatisfactory partner relations, difficulties in reaching orgasm, and concern about urine leakage during sexual activity^20^. Pelvic floor muscles play an important role in producing involuntary contractions, facilitating blood flow to the genital organs and providing better sensitivity during sexual activity^21,22^. A study of 106 women with hypoactive sexual desire disorder reported that group cognitive behavioral therapy contributed significantly to the improvement of sexual function^23^. Therefore, health professionals should provide information on various therapeutic alternatives, including pelvic floor muscle training and exercises, periodic voluntary contractions of pelvic muscles (Kegel exercises) with or without vaginal loads, electrical stimulation, and biofeedback therapy, to contribute to women's quality of life in the prevention and treatment of sexual dysfunction related to pelvic floor problems.

Since the participants were recruited from only one hospital, the results cannot be generalized to the country as a whole, but our results can be generalized to the province as the hospital is the largest single training and research hospital in the province where it is located and the urogynecology outpatient clinic receives a large number of patients from neighboring provinces and districts.

## CONCLUSION

Women with UI have poor sexual function. Pelvic organ prolapse distress, colorectal-anal distress, and urinary distress are important associated factors that negatively affect the sexual function of women with UI. Colorectal-anal distress was also found to be an important associated risk factor negatively affecting women's sexual function. Our findings suggest that healthcare professionals should thoroughly assess patients seeking care for UI for pelvic organ prolapse distress, colorectal-anal distress, and urinary distress and inquire about sexual function status.

## ETHICAL APPROVAL

At the outset, written approvals were obtained from the Non-Invasive Ethics Committee, Faculty of Health Sciences, Selcuk University (Issue: #2022/1168), and Aksaray Education and Research Hospital to conduct the study (Institution approval number: #:#E-93276592-448.01.01-205918970).

## Data Availability

In accordance with the guidelines of the Institutional Review Board (IRB) of Selcuk University, Faculty of Health Sciences, we are unable to share data of our current study because sharing data of this study compromises ethical standards or legal requirements.
